# AI-powered discovery of a novel p53-Y220C reactivator

**DOI:** 10.3389/fonc.2023.1229696

**Published:** 2023-08-01

**Authors:** Shan Zhou, Dafei Chai, Xu Wang, Praveen Neeli, Xinfang Yu, Aram Davtyan, Ken Young, Yong Li

**Affiliations:** ^1^ Department of Medicine, Section of Epidemiology and Population Sciences, Dan L Duncan Comprehensive Cancer Center, Baylor College of Medicine, Houston, TX, United States; ^2^ Atomwise Inc., San Francisco, CA, United States; ^3^ Hematopathology Division and Department of Pathology, Duke University Medical Center, Durham, NC, United States

**Keywords:** mutant p53, Y220C, artificial intelligence, small molecule inhibitors, apoptosis

## Abstract

**Introduction:**

The *p53-Y220C* mutation is one of the most common mutations that play a major role in cancer progression.

**Methods:**

In this study, we applied artificial intelligence (AI)-powered virtual screening to identify small-molecule compounds that specifically restore the wild-type p53 conformation from p53-Y220C. From 10 million compounds, the AI algorithm selected a chemically diverse set of 83 high-scoring hits, which were subjected to several experimental assays using cell lines with different p53 mutations.

**Results:**

We identified one compound, H3, that preferentially killed cells with the *p53-Y220C* mutation compared to cells with other p53 mutations. H3 increased the amount of folded mutant protein with wild-type p53 conformation, restored its transcriptional functions, and caused cell cycle arrest and apoptosis. Furthermore, H3 reduced tumorigenesis in a mouse xenograft model with *p53-Y220C*-positive cells.

**Conclusion:**

AI enabled the discovery of the H3 compound that selectively reactivates the p53-Y220C mutant and inhibits tumor development in mice.

## Highlights

- Y220C is one of the top p53 hotspot mutations
- A small compound H3 is discovered by an AI-powered approach
- H3 reactivates the p53-Y220C mutant and inhibits tumorigenesis

## Introduction

Tumor protein 53 (*TP53*) gene encodes tumor suppressor p53, a tetrameric transcription factor capable of binding to a defined DNA sequence, to regulate genes involved in diverse cellular processes, including the cell cycle, apoptosis, senescence, DNA repair, and metabolism (1–3). Collectively, these processes prevent cancer initiation and progression. p53 acts as a cellular stress sensor (4). Under normal conditions, p53 protein levels remain low via constant proteasomal degradation by the major E3 ligase, MDM2 (5). Upon activation, the tetrameric form of p53 binds to DNA and recruits the transcriptional machinery components to activate various anti-proliferative processes. In acute DNA damage, p53 promotes G1 cell cycle arrest to facilitate DNA repair or apoptosis to eliminate the damaged cells (6). The primary transcriptional targets of wild-type (WT) p53 are the cyclin-dependent kinase inhibitor 1A, which encodes p21 and mediates the G1 phase block, MDM2, which creates a negative feedback loop, and pro-apoptotic Bcl-2 family proteins (Bcl2-binding component 3, Bcl-2-associated X [BAX], and NADPH oxidase activator) (7).

Although *TP53* is known as the most mutated gene in cancer, it is considered to be undruggable. To date, only a few drugs have reached advanced clinical trial phases and no drugs targeting mutant p53 have been approved in the United States and Europe. However, many promising approaches have been developed to restore p53 activity in mutated cells. Several stabilizers, including CP-31398, p53 reactivation with the induction of massive apoptosis 1 (PRIMA-1), APR-246, RITA, PEITC, NSC319726, Chetomin, ReACp53, and pCAPs, have been developed to restore mutant p53 to its active form (8, 9). CP-31398, the first compound capable of reactivating mutant p53, was identified by Pfizer using a synthetic compound library screen (10). CP-31398 rescues mutant p53 conformation and stabilizes WT p53 conformation and prevents its degradation by reducing its ubiquitination, leading to high levels of transcriptionally active p53 (11). However, CP-31398 induces non-specific toxicity by intercalating with DNA and upregulating BAX expression in a p53 independent manner (12). A novel compound, PRIMA-1, was identified from a library of low-molecular-weight compounds (NCI Diversity Set) (13). PRIMA-1 and its potent methylated derivative, APR246 (eprenetapopt or PRIMA-1^Met^), restore WT function in the p53 mutants, R175H and R273H. APR-246 has shown positive results in phase I/II clinical trials (14), but in a recent phase III multicenter randomized trial (NCT03745716), APR-246 in combination with azacytidine failed to show an enhanced effect compared with azacytidine alone. Arsenic trioxide (ATO) is an effective p53 stabilizer capable of stabilizing 19 different structural p53 mutants (15). ATO targets mutant p53 and restores its transcriptional activity to inhibit tumor growth *in vivo*. Recently, Lu and colleagues used ATO to screen potential targets within the most common 800 missense mutations of p53 and identified 390 mutations that were rescuable by ATO (16). Moreover, several clinical trials on the roles of ATO in p53 mutant tumors are currently underway.

The Cancer Genome Atlas Pan-Cancer analyses revealed *TP53* as the most commonly mutated gene in human cancers (17). Patients with p53-mutant tumors usually have a worse prognosis and respond poorly to treatment (18). Most somatic missense mutations occur in the central DNA-binding domain (DBD) of p53, with 30% mutational hotspot residues affecting DNA binding and thermodynamic stability (19). R175, R248, R273, and R282 are the most commonly mutated codons (20). p53 mutants are classified as structural and DNA contact mutants. Structural mutants (R175H, R249S, G245S, and Y220C) reduce protein thermostability, leading to protein aggregation at physiological temperatures. *Y220C* is the ninth most common p53 mutation, accounting for an estimated 125,000 new cancer cases annually. It creates a narrow, well-defined surface pocket on the DBD surface that destabilizes the protein by 3.0–4.5 kcal/mol and reduces its melting temperature by approximately 8−9°C from 45°C (WT), leading to rapid denaturation and aggregation at body temperature (21). p53 aggregation in the form of amyloid oligomers and fibrils renders the p53 protein unable to bind to DNA for tumor suppression (13). Owing to the surface cavity created by this large-to-small mutation, *Y220C* is a suitable target site for structure-based drug design to restore p53 function in tumors.

**Figure 1:**
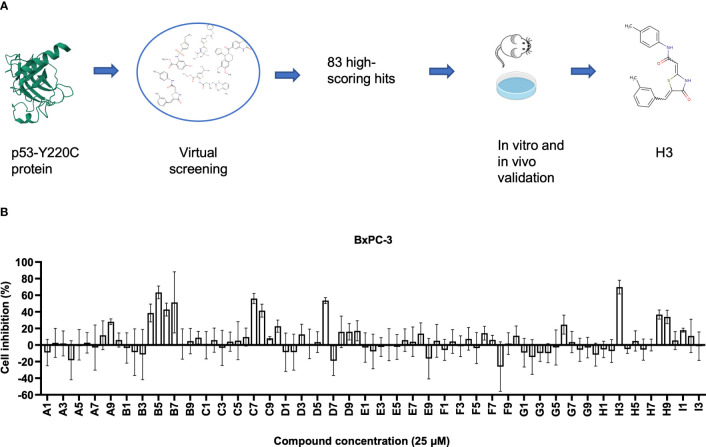
Identification small compounds targeting p53-Y220C. **(A)** Workflow of artificial intelligence (AI)-powered drug screening. **(B)** Cell viability normalized to the vehicle control of cell lines treated for 48 h with the indicated compounds. Error bars represent the mean ± standard deviation (SD). p53-Y220C protein 2D image was obtained from the Research Collaboratory for Structural Bioinformatics Protein Data Bank (RCSB PDB) with the ID of 6GGB (22).

In this study, we screened compounds that can restore the function of the p53-Y220C mutant using artificial intelligence (AI; **Figure 1A**). We identified a small molecule, with a code name H3 (N-(4-methylphenyl)-2-{5-[(3-methylphenyl)methylidene]-4-oxo-1,3-thiazolidin-2-ylidene}acetamide), that selectively reactivated p53-Y220C, rescued its transcription activity, and inhibited tumor development in mice.

## Materials and methods

### AI algorithm

AtomNet from Atomwise uses deep convolutional neural networks (DCNNs) and learns optimal model parameters to predict the binding of small molecules to a protein in a robust and efficient manner (23–28). The DCNNs are similar to those employed in image recognition and computer vision technologies but adapted for structure-based drug design and discovery. Trained on large data sets that comprise several thousand different protein structures and millions of protein-ligand binding affinity measurements (K_D_ and IC_50_ values), these DCNNs systemically and efficiently learn optimal model parameters for performing robust predictions and finding small molecules that specifically bind to a protein of interest, and not to other proteins in the same family.

### Reagents

Compounds used for screening were provided by Enamine Ltd (Monmouth Junction, NJ, USA) with over 90% purity. For *in vitro* assays, the compounds were dissolved in dimethyl sulfoxide (Sigma-Aldrich, St Louis, MO, USA). PhiKan083 (PK083) was purchased from Sigma-Aldrich and dissolved in ultrapure water. For *in vivo* experiments, the compounds were dissolved in corn oil (Sigma-Aldrich).

### Cell culture

Human lung cancer (H1993, H1975, and H1299), pancreatic cancer (BxPC-3), and murine melanoma (B16F10) cell lines were purchased from the American Type Culture Collection (Manassas, VA, USA). Human gastric cancer (NUGC-3) cell line was purchased from Sekisui XenoTech (Kansas City, KS, USA). Human hepatocellular carcinoma (Huh7) cell line was purchased from Creative Biolabs (Shirley, NY, USA). Human ovarian cancer (Cov362) cell line was purchased from Sigma-Aldrich. Human pancreatic cancer (PANC-1) and osteosarcoma (Saos-2) cell lines were obtained from the Molecular and Cellular Biology Tissue Culture Core Laboratory at the Baylor College of Medicine (Houston, TX, USA). BxPC-3, H1993, H1975, and H1299 cells were cultured in the Roswell Park Memorial Institute-1640 medium (Gibco, Grand Island, NY, USA) supplemented with 10% fetal bovine serum (FBS; Gibco) and 1% penicillin and streptomycin (P/S; Gibco). PANC-1, Huh7, Cov362, Saos-2, 293T, MC38, and B16F10 cells were cultured in the Dulbecco’s modified Eagle’s medium (Gibco) supplemented with 10% FBS and 1% P/S. All cell lines were confirmed to be mycoplasma-negative and incubated at 37°C with 5% CO_2_.

### Cell line construction

To generate *TP53* knockout (KO) cell lines, we performed ribonucleoproteinelectroporation genome editing experiments on BxPC-3 and NUGC-3 cells. Chemically synthesized Alt-R-modified single guide RNAs (sgRNAs) (sg1: CCATTGTTCAATATCGTCCG; sg2: TCCACTCGGATAAGATGCTG) that target *TP53* and Alt-R S.p. HiFi Cas9 Nuclease V3 were purchased from Integrated DNA Technologies (Coralville, IA, USA).

Trp53 Mouse Gene Knockout Kit (OriGene, Rockville, MD, USA) was used to generate *Trp53-*KO MC38 and B16F10 cells. Briefly, 1 μg sgRNAs (sg: ATCCGACTGTGACTCCTCCA) with the *Trp53* target DNA sequences and 1 μg linear donor DNA containing EF1a-GFP-P2A-Puro were used. Subsequently, MC38 or B16F10 cells (1 × 10^6^) were transfected with the sgRNA/linear donor DNA complexes using a 4D-Nucleofector system and the SF Cell Line 4D-Nucleofector X Kit (Lonza, Basel, Switzerland). Puromycin selection was initiated approximately three weeks after transfection. *Trp53*-KO cells were sorted via flow cytometry three days after puromycin selection using the BD FACSAria II cell sorter. Western blotting was performed to evaluate *Trp53*-KO, and the stability of the KO was confirmed via flow cytometry and western blotting analysis three weeks after sorting.

To generate *p53-Y220C-*overexpressing cells, B16F10 and MC38 *Trp53*-KO cells were transfected with pUltra-hot-*p53-Y220C* using a 3^rd^ lentiviral transduction system. A total of 20 μg of plasmid DNA was used for the transfection of 293T cells: 3.5 μg of the envelope plasmid pCMV-VSV-G, 6.5 μg of packaging plasmid pMDLg/pRRE and pRSV-Rev, and 10 μg of pUltra-hot-p53-Y220C plasmid. B16F10-*p53-Y220C* and MC38-*p53-Y220C* cells were sorted via flow cytometry seven days after transduction.

### Cell viability assay

Cells were seeded at a density of 3,000 cells/well in a 96-well plate and incubated overnight. For the initial 1-dose (25 μM) screening. BxPC-3 cells were treated with compounds at 25 µM for 48 h. Next, 20 µL of CellTiter 96 Aqueous One Solution Reagent (Promega Corporation, Madison, WI, USA) was added to each well. After incubation for 4 h, the absorbance was recorded at 490 nm using a CLARIOstar Plus spectrophotometer (BMG LABTECH Inc., Cary, NC, USA). For the second-round screening. BxPC-3, NUGC-3, Cov362, and Huh7 cells were treated with compounds at a concentration of 10 µM 72 h. Then, 100 μL of CellTiter-Glo 2.0 Reagent (Promega Corporation) was added to each well to measure the fluorescence intensity at 485–500 nm_Ex_/520–530 nm_Em_ using CLARIOstar Plus (BMG LABTECH Inc.). For half-maximal inhibitory concentration (IC_50_) detection, BxPC-3, NUGC-3, Cov362, Huh7, PANC-1, H1993, H1975, H1299, and Saos-2 cellswere treated with various concentrations (5, 10, 20, 50, 75, and 100 µM) of compounds for 72 h. BxPC-3, BxPC-3-TP53-KO, NUGC-3, and NUGC-3-TP53-KO cells were treated with various concentrations (5, 10, 12.5, 15, 17.5, 20, 22.5, 25, 30, and 50 µM). Then, 100 μL of CellTiter-Glo 2.0 Reagent (Promega Corporation) was added to each well to measure the fluorescence intensity at 485–500 nm_Ex_/520–530 nm_Em_ using CLARIOstar Plus (BMG LABTECH Inc.). All assays were performed in triplicate.

### RNA isolation and quantitative reverse transcription-polymerase chain reaction (qRT-PCR)

Total RNA was extracted and purified using the RNeasy Plus Micro Kit (Qiagen, Valencia, CA, USA). The RNA was then reverse-transcribed using the LunaScript RT SuperMix Kit (New England Biolabs, Beverly, MA, USA), according to the manufacturer’s protocol. For qPCR, the FastStart Universal SYBR Green Master (Rox) kit (Roche Applied Science, Mannheim, Germany) was used in triplicate for each sample. Each reaction was run in final volume of 25 µL containing 2.5 µL first-strand cDNA, 1 µL (5 µM) of each primer (Sigma-Aldrich), 12.5 µL of 2X Fast Start Universal SYBR Green Master (ROX), and 8 µL of distilled water. Thermal cycling conditions were set according to the manufacturer’s instructions, and reactions were performed using a QuantStudio 7 Pro qPCR machine (Thermo Fisher Scientific, Waltham, MA, USA). The comparative threshold cycle (Ct) method was used to analyze the gene expression level, and the results were obtained using ΔΔCT method. The primer sequences used were as follows: *MDM2* forward 5′- GAATCATCGGACTCAGGTACATC-3′ and reverse 5′- TCTGTCTCACTAATTGCTCTCCT-3′ (167-bp product); *Puma* forward 5′- GCCAGATTTGTGAGACAAGAGG-3′ and reverse 5′- CAGGCACCTAATTGGGCTC-3′ (136-bp product); *p21* forward 5′- TGTCCGTCAGAACCCATGC -3′ and reverse 5′- AAAGTCGAAGTTCCATCGCTC-3′ (139-bp product); and glyceraldehyde 3-phosphate dehydrogenase (*GAPDH*) forward 5′- GGAGCGAGATCCCTCCAAAAT-3′ and reverse 5′- GGCTGTTGTCATACTTCTCATGG-3’ (197-bp product). Amplification of the housekeeping gene *GAPDH* was used as a reference for the normalization of gene expression levels.

### Western blotting

BxPC-3, NUGC-3, Cov362, Huh7, B16F10-p53-Y220C, and MC38-p53-Y220C cells were harvested, washed twice with phosphate-buffered saline (PBS; Gibco), and lysed using the radioimmunoprecipitation assay buffer (Thermo Fisher Scientific) containing a cocktail of protease and phosphatase inhibitors (Thermo Fisher Scientific). Protein concentrations were determined using the Pierce BCA Protein Assay Kit (Thermo Fisher Scientific). Protein lysates were then subjected to Tris-glycine sodium dodecyl sulfate-polyacrylamide gel electrophoresis (4–20% polyacrylamide; Mini-PROTEAN Precast Gels; Bio-Rad, Hercules, CA, USA) and transferred to 0.2-μm pore size hydrophobic polyvinylidene difluoride membrane (EMD Millipore Corp, Billerica, MA, USA). Blotting was performed using primary antibodies directed against p53 and other proteins and secondary antibodies coupled with horseradish peroxidase (HRP)-conjugated corresponding secondary antibodies. Antibodies were purchased from Santa Cruz Biotechnology (Dallas, TX, USA; p53, DO-1, #sc-126) and Cell Signaling Technology (Danvers, MA, USA; MDM2, E3G5I, #51541; PUMA, E2P7G, #98672; p21, E2R7A, #37543; GAPDH, 14C10, #2118; Anti-mouse IgG, HRP-linked Antibody, #7076; Anti-rabbit IgG, HRP-linked Antibody, #7074). The signal was visualized using the SuperSignal West Dura Extended Duration Substrate (Thermo Fisher Scientific).

### Immunoprecipitation (IP)

BxPC-3, NUGC-3, Cov362, and Huh7 ells were treated with the indicated concentrations of compounds for 24 h, harvested, and washed twice with PBS. Cells were lysed with the IP lysis buffer (Thermo Fisher Scientific), sonicated, and incubated on ice for 30 min. The lysates were centrifuged at 14,000 rpm for 20 min and the resulting supernatant was adjusted to a final concentration of 1 mg/mL using the IP lysis buffer. To immunoprecipitate the proteins, 500 µL of the supernatant was added to 20 µL of the protein A/G mix magnetic beads (EMD Millipore Corp.) along with 2 µg of PAb1620 (EMD Millipore Corp.; #MABE339) or PAb240 (Abcam, Cambridge, MA, USA; #ab26) antibody. The mixture was rotated overnight at 4 °C. The magnetic beads were washed thrice with 1 mL IP lysis buffer, suspended in 45 µL of IP lysis buffer, and boiled for 10 min with 15 µL of 4 × Invitrogen NuPAGE LDS Sample Buffer (Thermo Fishers Scientific). The resulting mixture was subjected to western blotting using the VeriBlot for IP Detection Reagent (Abcam, #ab131366) as the HRP-conjugated secondary antibody.

### Cell cycle analysis

BxPC-3, NUGC-3, Cov362, Huh7, B16F10-p53-Y220C, and MC38-p53-Y220C cells were treated with the indicated compounds, collected, and washed with PBS. Cells were fixed overnight with 70% ice-cold ethanol at –20°C and washed twice with PBS. Fixed BxPC-3, NUGC-3, Cov362, and Huh7 cells were stained with the BD PI/RNase Staining Buffer (BD Biosciences, Franklin Lakes, New Jersey, USA). B16F10-p53-Y220C and MC38-p53-Y220C cells were stained with 7-amino-actinomycin D (7-AAD; BioLegend, San Diego, CA, USA) and analyzed using a Northern Lights flow cytometer (Cytek Biosciences, Fremont, CA, USA).

### Apoptosis analysis

Apoptosis of BxPC-3, NUGC-3, Cov362, and Huh7 cells was analyzed using the FITC Annexin V Apoptosis Detection Kit I (BD Biosciences), whereas that of B16F10-p53-Y220C and MC38-p53-Y220C cells was analyzed using the APC Annexin V Apoptosis Detection Kit with 7-AAD (BioLegend), according to the manufacturers’ instructions. Flow cytometry data were analyzed using the FlowJo V10 software (FlowJo LLC, Ashland, OR, USA).

### Mouse xenograft tumor model

NOD.Cg-Prkdcscid Il2^rgtm1Wjl/SzJ^ (NSG) mice (age: 6–8 weeks) were purchased from Jackson Laboratory (Bar Harbor, ME, USA) and maintained at the Baylor College of Medicine Animal Facility. NUGC-3 cells (1 × 10^6^ cells/mouse) were subcutaneously injected into the right flank of mice. Eight days post-inoculation, 10 mice were randomly grouped and treated with either an intraperitoneal injection of vehicle (DMSO) or H3 (5 mg/kg) every other day until humane endpoints were reached. For H9, we used 9 × 10^5^ NUGC-3 cells/mouse for inoculation and 25 mg compound/kg for treatment. Humane endpoints were defined as tumor volumes reached 1.5 cm, tumor burden was greater than or equal to 10% of the normal body weight of the animal, or severe tumor necrosis. Tumor volume was calculated using the following equation: V (mm3) = (length × width^2^)/2. The tumor burden and mouse weight were monitored every other day. All procedures were performed with the approval of the Institutional Animal Care and Use Committee of the Baylor College of Medicine.

### Immunohistochemistry (IHC) and TdT-mediated dUTP nick-end labeling (TUNEL) assay

Tumor tissues were fixed in 10% formaldehyde and subjected to hematoxylin and eosin (H&E) staining, IHC, and TUNEL assay by the Human Tissue Acquisition and Pathology (HTAP) Core Lab at the Baylor College of Medicine as previously described (29).

### Statistical analysis

Statistical analyses were performed using the Prism 8 software (GraphPad Software, San Diego, CA, USA). Unpaired two-tailed Student’s *t*-test, one-way analysis of variance (ANOVA), or two-way ANOVA were used for significant differences. Data are represented as the mean ± standard deviation (SD). Significant differences between two groups are indicated by asterisks (*p < 0.05, **p < 0.01, ***p < 0.001, and **** *p* < 0.0001).

## Results

### Compound screening

Using the AtomNet algorithm (23–28), we virtually screened around 10 million commercially available compounds against a unique elongated surface crevice from the high-resolution structure (PDB ID: 6GGB, 1.32 Å) of p53-Y220C mutant with a known stabilizer, PK9318 (22). This is a well-known strategy for counteracting the effect of the *Y220C* mutation as the described pocket is not present in WT p53. We obtained a chemically diverse set of 83 high-scoring predicted hits with purity > 90% (**Supplemental Table 1**). We conducted an initial 1-dose (25 μM) screening of these compounds using BxPC-3 cells containing p53-Y220C and found several compounds (including B5 and H3) with viability inhibition potency (**Figure 1B**). We then performed a second 1-dose (10 μM) screening using several cell lines harboring the *p53-Y220C* mutation (BxPC-3, NUGC-3, Cov362, and Huh7). The top five compounds (B4, B5, B7, H3, and H9) were selected for further validation (**Figures S1A–D**).

**Figure 2:**
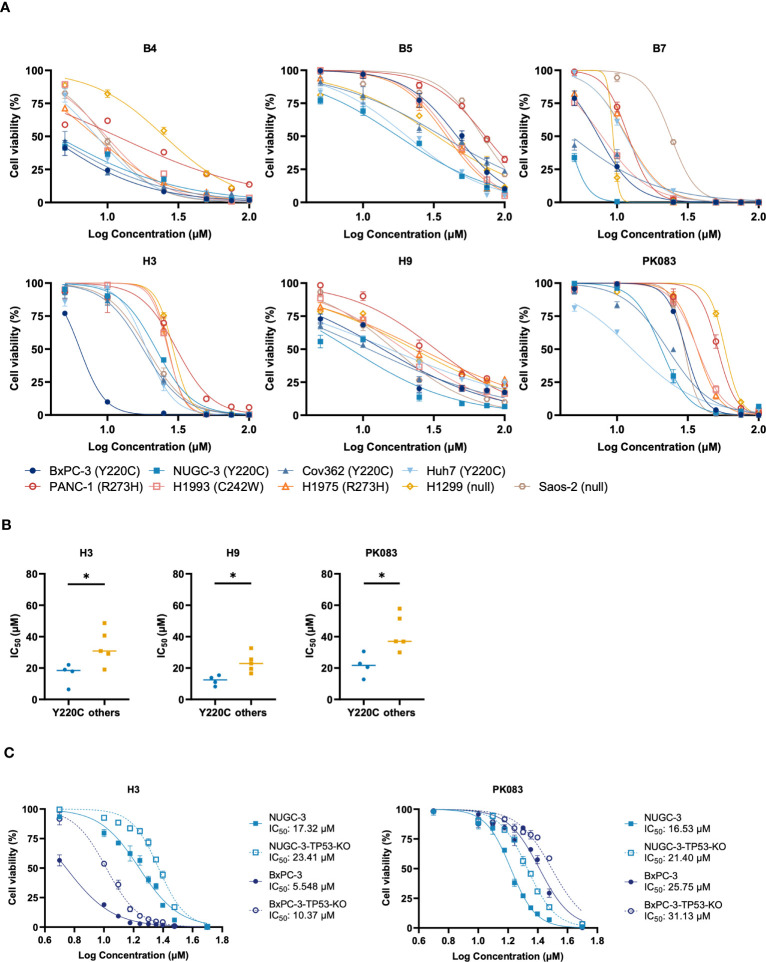
Y220C-specificity of compounds involved in cell proliferation inhibition. **(A)** Viability of indicated cell lines expressing endogenous p53 mutant treated with B4, B5, B7, H3, H9, or PK083 for three days. Half-maximal inhibitory concentration (IC_50_) values were calculated using non-linear curve fitting with GraphPad Prism 8.0. **(B)** Dots represent the calculated IC_50_ values in cell lines with *p53-Y220C* or other mutations. **(C)** Mutant p53-Y220C in NUGC-3 and BxPC-3 cells was knocked out using the clustered regularly interspaced palindromic repeat (CRISPR)/CRISPR-associated protein 9 (Cas9) system, and viability was assessed using the cell viability assay. Error bars in **(A, C)** represent the mean ± SD. *p* values were calculated using two-tailed *t*-tests. *p < 0.05.

We assessed the ability of the candidate compounds with concentrations ranging from 5.0-100 μM to inhibit the growth of cell lines with *p53-Y220C* mutation (**Figure 2A**). This dose-response assay also resolved some discrepancies in inhibiting cell viability with 10 μM and 25 μM. Compared with the vehicle control, B4, B7, H3, and H9 compounds exhibited stronger killing activity against cancer cell lines with *Y220C* mutation. Cell lines with other p53 mutations exhibited resistance to these compounds, as evidenced by their IC_50_ values. To determine the correlation between *TP53* mutation status and compound-induced growth inhibition, we compared the IC_50_ values between cell lines with the *Y220C* mutation and those with other p53 mutations (**Figures 2B**; **S2**). We observed that the IC_50_ values of H3, H9, and PhiKan083 (PK083) were lower in cell lines with the *Y220C* mutation (**Figure 2B**). To confirm the involvement of the *p53-Y220C* mutation in the growth inhibition mediated by these compounds, we knocked out *TP53* in these cell lines using the clustered regularly interspaced palindromic repeat (CRISPR)/CRISPR-associated protein 9 system. We found *TP53* KO increased the IC_50_ values of H3 in both BxPC-3 and NUGC-3 cell lines (**Figure 2C**). These results indicate that the growth inhibition of BxPC-3 and NUGC-3 cells by H3 is at least partially mediated by *p53-Y220C*.

### H3 induces p53 target gene expression in p53-Y220C cells

**Figure 3:**
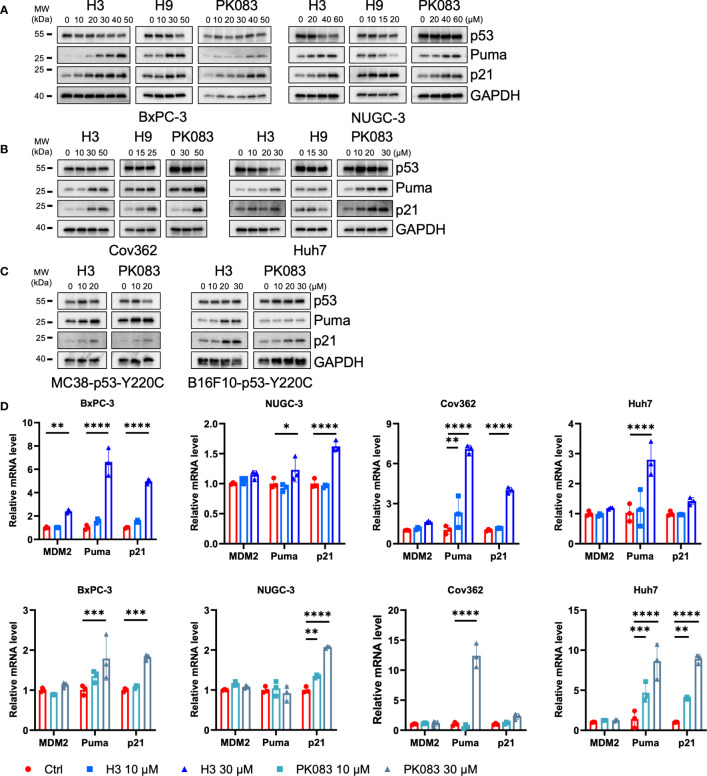
H3 activates p53 target gene expression. **(A–C)** Immunoblotting to determine the protein levels of the indicated p53 targets after H3, H9, and PK083 treatment for 24 h. **(D)** Quantitative polymerase chain reaction (qPCR) determination of the mRNA levels of the indicated p53 targets after H3 and PK083 treatment for 24 h. Quantitative reverse transcription (qRT)-PCR analyses results were normalized using glyceraldehyde 3-phosphate dehydrogenase (*GAPDH*) as an internal control gene. The fold-change in each gene was calculated using the 2^−ΔΔ^ *^CT^* method. Error Bars in **(D)** represent the mean ± SD. *p* values were calculated using two-way ANOVA analysis followed by Dunnett’s multiple-comparison test. *p<0.05, **p<0.01, ***p<0.001, and ****p<0.0001.

To test the ability of the candidate compounds to reactivate the endogenous mutant p53-Y220C, we treated a panel of *p53-Y220C*-positive cancer cell lines with H3, H9, and PK083 and monitored the protein levels of p53, Puma, and p21 (**Figures 3A, B**). Following H3 treatment, BxPC-3, NUGC-3, Cov362, and Huh7 cells exhibited increased Puma and p21. After H9 treatment, BxPC-3, NUGC-3, and Cov362 cells exhibited p21 upregulation, BxPC-3 and Cov362 cells exhibited Puma upregulation. Following PK083 treatment, all cell lines exhibited potent induction of Puma and p21. Next, we constructed *Trp53* KO cells using MC38 and B16F10 cells and used lentiviruses to overexpress human *p53-Y220C* in these two cell lines. In MC38-p53-Y220C cells, H3 treatment induced potent expression of Puma, and p21, which was comparable to that induced by PK083 treatment (**Figure 3C**). In B16F10-p53-Y220C cells, H3 induced higher expression of Puma and p21 than PK083 (**Figure 3C**). We determined the mRNA expression levels of p53 transactivation targets in the four *p53-Y220C*-positive cell lines treated with H3 or PK083. In H3-treated cells, both compounds upregulated the levels of representative p53 targets, MDM2, Puma, and p21, in a dose-dependent manner (**Figure 3D**). These findings indicate that H3 upregulates p53 transactivation in cancer cells expressing *p53-Y220C*.

### H3 induces WT p53 structural conversion from p53-Y220C

**Figure 4:**
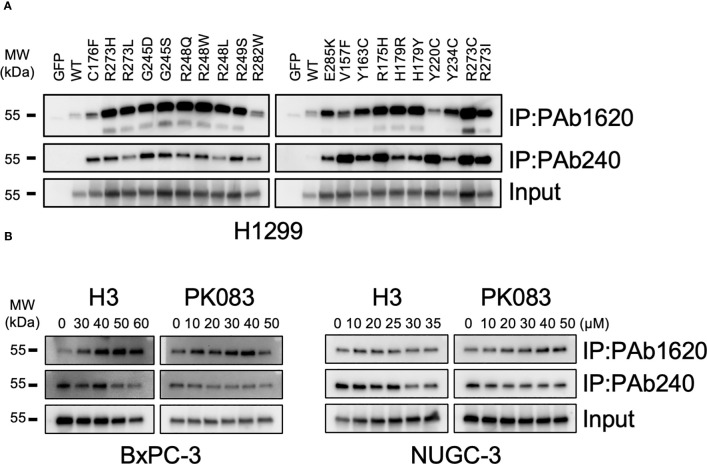
Structural conversion induced by H3. **(A)** PAb1620 and PAb240 immunoprecipitation (IP) for H1299 cells exogenously expressing the indicated p53 mutants. **(B)** PAb1620 and PAb240 IP for cells endogenously expressing the p53-Y220C mutant treated with H3, H9, and PK083 for 24 h. Immunoblotting was performed using the anti-p53 DO-1 antibody after IP.

PAb1620 antibody prefers folded WT p53, whereas the PAb240 antibody specifically recognizes an epitope of the unfolded DBD from p53 mutants. To evaluate the specificity of these antibodies, we constructed expression vectors for the 20 most common somatic p53 mutations that were expressed in the *p53*-null human non-small cell lung cancer cell line, H1299. PAb1620 and PAb240 were used to immunoprecipitate soluble cell lysates, which were then subjected to western blotting using a pan-p53 antibody (DO-1). Under overexpression conditions in H1299 cells, PAb240 did not recognize WT p53 but bound to p53-Y220C; PAb1620 immunoprecipitated WT p53 and most p53 mutants, but its binding with p53-Y220C was weak (**Figure 4A**).

We treated BxPC-3, NUGC-3, Cov362, and Huh7 cells with various dosages (10–60 μM) of H3, H9, and PK083. In BxPC-3 and NUGC-3 cells treated with H3 and PK083, the PAb1620 epitope levels increased, whereas the PAb240 epitope levels decreased in a dose-dependent manner (**Figure 4B**). In NUGC-3 and Cov362 cells treated with H9, no significant changes were observed in PAb1620 and PAb240 epitope levels (**Figure S3**). In contrast, PAb240 epitope levels decreased after H3 treatment (**Figure 4B**). In Huh7 cells, a significant decrease in both PAb1620 and PAb240 epitope levels was observed after H9 treatment (**Figure S3**). PK083 induced a dose-dependent increase in the expression of PAb1620 epitope (**Figure 4B**). These results indicate that H3 promotes a conformational change from mutant p53-Y220C to WT p53.

### H3 induces apoptosis and cell cycle arrest in *p53-Y220C*-expressing cell lines

**Figure 5:**
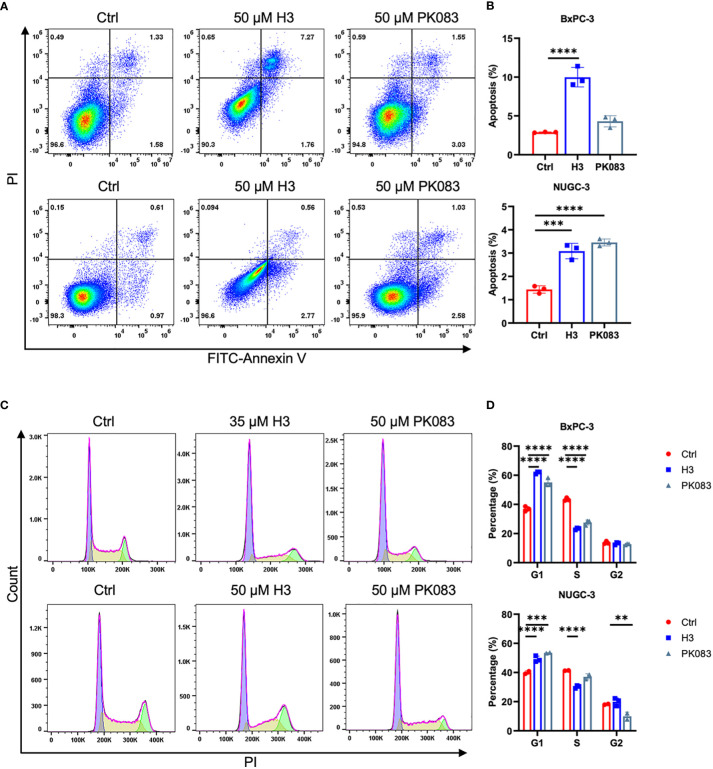
H3 induces cycle arrest and apoptosis in p53-Y220C mutant cells **(A)** Flow cytometric plots of annexin V vs. propidium iodide (PI) staining in BxPC-3 cells after treatment with 50 μM H3 or 50 μM PK083 for 42 h, and NUGC-3 cells after treatment with 50 μM H3 or 50 μM PK083 for 24 h. **(B)** From the histograms, the proportion of cell apoptosis were statistically analyzed. *p* values were calculated by one-way ANOVA followed by Dunnett’s multiple-comparison test. **(C)** Cell cycle analysis via PI staining followed by flow cytometry. BxPC-3 cells after treatment with 35 μM H3 or 50 μM PK083 for 24 h, and NUGC-3 cells after treatment with 50 μM H3 or 50 μM PK083 for 24 h. **(D)** From the histograms, the proportion of cell cycle distribution were statistically analyzed. *p* values were calculated by two-way ANOVA followed by Dunnett’s multiple-comparison test. Error Bars in **(B, D)** represent mean ± SD. **p < 0.01, ***p < 0.001, and ****p < 0.0001.

We studied the effects of H3 on cell apoptosis and cell cycle using flow cytometry in various cell lines expressing *p53-Y220C* (**Figures 5A–D**; **S4A–H**). H3 treatment increased the apoptosis in BxPC-3, NUGC-3, Cov362, and Huh7 cells as well as in B16F10-p53-Y220C and MC38-p53-Y220C cells. H3 treatment induced a significantly higher proportion of cells in the G1 phase in BxPC-3 and NUGC-3 cells than in the S phase in Cov362 cells. H3 treatment did not significantly affect the distribution of cells in G1 and S phases in Huh7 cells. There was a significant increase in the proportion of cells in the G1 phase in B16F10-p53-Y220C cells after H3 treatment, whereas no such difference was observed in the distribution of cells in the G1 and S phases in MC38-p53-Y220C cells (**Figures S4G-H**). Similar to H3 treatment, PK083 treatment promoted apoptosis in NUGC-3, Cov362, Huh7, B16F10-TP53-Y220C, and MC38-p53-Y220C cells; PK083 induced a higher proportion of cells in G1 phase in BxPC-3 and NUGC-3 cells and a higher proportion of cells in S phase in Cov362 cells. However, PK083 treatment did not significantly affect the distribution of cells in G1 and S phases in Huh7, B16F10-p53-Y220C, and MC38-p53-Y220C cells (**Figures S4C-D, G-H**). These results suggest that H3 enhances apoptosis and induces cell cycle arrest in cells expressing *p53-Y220C*.

### H3 inhibits the growth of mouse xenograft tumors

**Figure 6:**
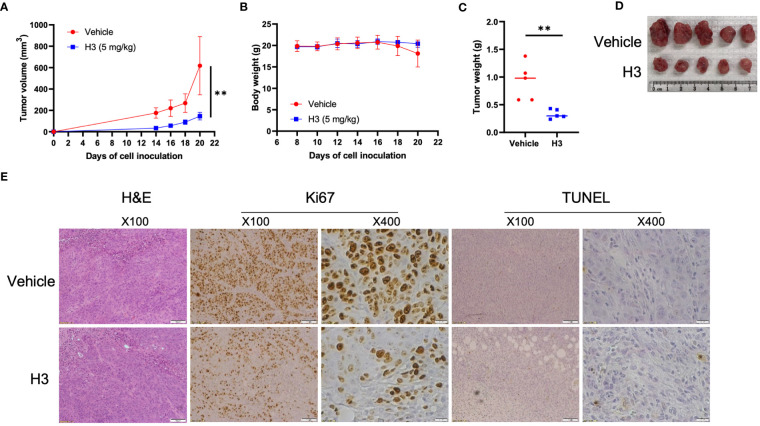
H3 inhibits NUGC-3 tumor growth in mice. **(A)** Immunodeficient NSG mice were subcutaneously injected with 1 × 10^6^ NUGC-3 cells on day 0. Eight days post-inoculation, 10 mice were randomly divided into two groups. Mice were treated with the vehicle control or 5 mg/kg H3 every other day until the first control mouse reached the humane endpoints. **(B)** Body weights of mice carrying NUGC-3 tumors after treatment. **(C)** Graphical quantification of the difference in tumor weights on day 20 in both groups. **(D)** Images of tumors excised from mice on day 20 in various treatment groups. **(E)** Representative tissue sections from two tumors after hematoxylin and eosin (H&E), Ki67, and TdT-mediated dUTP nick-end labeling (TUNEL) staining. Histological images are representative of five mice per group. Error Bars in **(A, B)** represent the mean ± SD. *p* values were calculated using two-tailed *t*-tests. **p < 0.01.

To investigate the *in vivo* effects of H3 and H9 on tumor growth, we used the NUGC-3 mouse xenograft model. We subcutaneously injected 1 × 10^6^ NUGC-3 cells into immunodeficient NSG mice on day 0 and visible tumors developed at the injection sites after eight days. Ten NSG mice were randomly divided into the vehicle control and compound treatment groups. We administered the vehicle control or H3 (5 mg/kg) every other day until the humane endpoint. Compared with the vehicle control, H3 treatment significantly decreased the tumor volume and weight but did not affect the animal body weight (**Figures 6A–D**). We also analyzed the expression of Ki67 in tumor tissues via IHC staining and found that tumor cell proliferation was significantly inhibited in H3-treated tumors. Furthermore, TUNEL staining revealed that the number of apoptotic tumor cells increased in the H3 treatment group than in the vehicle control group (**Figure 6E**). Interestingly, even at a high dose (25 mg/kg every other day), no significant differences in the tumor size, tumor weight, or body weight were observed between the H9 treatment and control groups (**Figures S5A–D**). These data indicate that H3 inhibits tumor growth and induces apoptosis in mouse xenografts expressing *p53-Y220C in vivo*.

## Discussion

Over 2,000 p53 somatic mutations have been identified in cancer, approximately 90% of which are missense mutations predominantly located in the DBD. The large number of *TP53* missense mutations makes them attractive targets for drug development. Several strategies for targeting p53 have been proposed. MDM2 is the primary E3 ligase that mediates p53 degradation; therefore, MDM2 inhibition may be used to target tumors with WT p53. Efforts to target mutant p53 have been ongoing for more than two decades; however, only a few molecules have reached the clinical trial stage (30). *p53-Y220C* mutation is the most common *p53* mutation outside of the DNA-binding surface, affecting approximately 1% of solid tumors (31). Several small-molecule compounds targeting p53-Y220C have been identified, including N-ethylcarbazole PK083 (32), pyrazole-based compound PK7088 (33), iodophenol derivative PK5196 (34), aminobenzothiazole derivative MB710 (35) and PK9318 (22). In silico screening revealed that PK083 binds to the Y220C pocket and increases the melting temperature of the mutant (32). PhiKan5196 was identified through halogen-enriched fragment library screening and was shown to induce apoptosis in Y220C-containing NUGC-3 cells (34). PK7088 reactivates p53-Y220C, leading to the growth inhibition, cell cycle arrest, and apoptosis of cancer cells (33). MB725 reduced the viability of p53-Y220C cancer cell lines and showed low toxicity in cell lines without p53-Y220C (35). PK9318 is the optimized version of PK083 that restores p53 signaling in the liver cancer cell line Huh7 with a homozygous *Y220C* mutation (22). PC14586, developed by PMC Pharma, is a small-molecule structural corrector that stabilizes the p53-Y220C protein in the WT p53 conformation, restoring p53 transactivation and tumor-suppressive function (36). Recently, KG13, the first p53 Y220C-specific covalent compound, was developed, which promotes Y220C thermal stability to WT p53 levels and activates p53 in a Y220C-dependent manner (37). However, only PC14586 is currently being used in clinical trials.

In this study, we used AI-aided virtual screening to identify novel p53-Y220C reactivators. For the 83 compounds selected by AI, we performed two rounds of cell viability and found five compounds with the most potent killing toward p53-Y220C cell lines compared to other lines. Our results showed that compounds H3 and H9 had significantly lower IC_50_ values in the p53-Y220C cell line, demonstrating their specificity toward the p53-Y220C mutant. To test the therapeutic potential of H3, we treated a series of cell lines with *p53-Y220C* with H3, and H9. We monitored target gene expression, p53 structural conformation, and its effects on the cell cycle and apoptosis. Both H3 and H9 promoted p53 target gene expression, whereas only H3 restored WT p53 conformation from p53-Y220C. In addition, H3 induced p53-Y220C-dependent cell cycle arrest and apoptosis. Treatment with H3 significantly inhibited tumor growth in the NUGC-3 xenograft NSG mouse model. Notably, the structure of H3 does not appear to share any chemophores with the reported compounds targeting p53-Y220 or other mutants, underscoring the potential of AI in exploring unknown chemical spaces.

This study has a few limitations. First, we did not have biophysical data to demonstrate H3 binding to p53-Y220C as we could not obtain soluble p53-Y220C or its DBD without four stabilizing mutations (M133L/V203A/N239Y/N268D) (22). Second, the IC_50_ value of H3 must be at a sub-nanomolar level for further in-depth study. Third, the toxicity of H3 remains unclear and should be investigated in future studies.

In summary, we used AI to discover a novel compound, H3, that selectively reactivated the p53-Y220C mutant and inhibited tumor development in mice. These results highlight the potential use of AI-powered drug screening to investigate individual p53 mutants in the future.

## Data availability statement

The original contributions presented in the study are included in the article/**Supplementary Material**. Further inquiries can be directed to the corresponding authors.

## Ethics statement

The animal study was reviewed and approved by IACUC, Baylor College of Medicine.

## Author contributions

SZ, DC, and YL: Conceived and designed the project; SZ, DC, and AD: Performed the project and analyzed the data; SZ, DC, XW, AD, PN, XY, KY, and YL: Contributed reagents, materials, and analysis tools and wrote, reviewed, and edited the manuscript. All authors contributed to the article and approved the submitted version.
