# Supplementary material for: AI-powered discovery of a novel p53-Y220C reactivator
Source: Front Oncol. 2023 Aug 1;13:1229696. doi: 10.3389/fonc.2023.1229696 (PMC10430779; doi:10.3389/fonc.2023.1229696)
Supplement: Supplementary file 1 [file DataSheet_1.pdf]

Supplementary Table and Figures

Supplemental Table 1

No	Code	MW	Systematic name	SMILES
1	A01	420.436	2-(1-methanesulfonyl-1,2,3,4-tetrahydroquinolin-6-yl)-2-oxoethyl 2-[(furan-2-yl)formamido]acetate	CS(N1C2=C(C=C(C(COC(CNC(C3OC=CC=3)=O)=O)=O)C=C2)CCC1)(=O)=O
2	A02	464.899	2-(6-amino-1,3-dimethyl-2,4-dioxo-1,2,3,4-tetrahydropyrimidin-5-yl)-2-oxoethyl (2S,3S)-2-[(4-chlorophenyl)formamido]-3-methylpentanoate	CC[C@@H]([C@@H](C(OCC(C1C(=O)N(C)C(=O)N(C)C=1N)=O)=O)NC(C1=CC=C(Cl)C=C1)=O)C
3	A03	275.322	carbamoyl(phenyl)methyl 5-methylthiophene-2-carboxylate	CC1SC(C(OC(C2=CC=CC=C2)C(N)=O)=O)=CC=1
4	A04	344.384	1-[2-(3-methyl-1-benzothiophen-2-yl)-2-oxoethyl]-3-(propan-2-yl)imidazolidine-2,4,5-trione	CC(N1C(=O)C(=O)N(CC(C2=C(C)C3=C(C=CC=C3)S2)=O)C1=O)C
5	A05	361.822	propan-2-yl 3-(2-chlorophenyl)-3-{[(pyridin-3-yl)carbamoyl]amino}propanoate	CC(OC(CC(C1=C(Cl)C=CC=C1)NC(NC1=CN=CC=C1)=O)=O)C
6	A06	347.405	4-benzyl 1-tert-butyl (2S)-2-(cyclopropylformamido)butanedioate	CC(OC([C@@H](NC(C1CC1)=O)CC(OCC1=CC=CC=C1)=O)=O)(C)C
7	A07	475.539	2-[1-(1,5-dimethyl-3-oxo-2-phenyl-2,3-dihydro-1H-pyrazol-4-yl)-2,5-dimethyl-1H-pyrrol-3-yl]-2-oxoethyl 1,3,5-trimethyl-1H-pyrazole-4-carboxylate	CC1N(C2C(=O)N(C3=CC=CC=C3)N(C)C=2C)C(C)=C(C(COC(C2=C(C)N(C)N=C2C)=O)=O)C=1
8	A08	330.338	N-[carbamoyl(phenyl)methyl]-3-(6-methyl-2,4-dioxo-1,2,3,4-tetrahydropyrimidin-5-yl)propanamide	CC1=C(CCC(NC(C2=CC=CC=C2)C(N)=O)=O)C(=O)NC(=O)N1
9	A09	454.334	benzyl 2-[(2-bromophenyl)formamido]-4-methanesulfonylbutanoate	CS(CCC(C(OCC1=CC=CC=C1)=O)NC(C1=C(Br)C=CC=C1)=O)(=O)=O
10	A10	335.373	N-({[cyclobutyl(4-fluorophenyl)methyl]carbamoyl}methyl)-2-acetamidoacetamide	CC(NCC(NCC(NC(C1=CC=C(F)C=C1)C1CCC1)=O)=O)=O
11	B01	471.516	3-(2-{[1-benzyl-5-(trifluoromethyl)-1H-1,3-benzodiazol-2-yl]sulfanyl}ethanesulfonyl)propanamide	NC(CCS(CCSC1N(CC2=CC=CC=C2)C2=C(C=C(C(F)(F)F)C=C2)N=1)(=O)=O)=O
12	B02	320.333	N-[2-(3,5-dimethylphenyl)-2-hydroxyethyl]-2,2-difluoro-2-(pyridin-2-yl)acetamide	CC1=CC(C(CNC(C(C2=NC=CC=C2)(F)F)=O)O)=CC(C)=C1
13	B03	440.308	methyl 2-{2-[(3-bromophenyl)methanesulfonyl]acetamido}-2-phenylacetate	COC(C(C1=CC=CC=C1)NC(CS(CC1=CC(Br)=CC=C1)(=O)=O)=O)=O
14	B04	389.468	methyl (2S)-3,3-dimethyl-2-({5-[(phenylcarbamoyl)amino]thiophen-2-yl}formamido)butanoate	COC([C@H](C(C)(C)C)NC(C1SC(NC(NC2=CC=CC=C2)=O)=CC=1)=O)=O
15	B05	341.402	methyl 2-(5-ethylthiophene-2-sulfonamido)-4-hydroxybenzoate	CCC1SC(S(NC2=C(C(OC)=O)C=CC(O)=C2)(=O)=O)=CC=1
16	B06	404.460	4-methoxy-3-nitro-N-[phenyl(thiophen-2-yl)methyl]benzene-1-sulfonamide	COC1=C(N(=O)=O)C=C(S(NC(C2=CC=CC=C2)C2SC=CC=2)(=O)=O)C=C1
17	B07	445.574	ethyl 2-[2-({[(2,3-dimethylphenyl)carbamoyl]methyl}(methyl)amino)acetamido]-5-(propan-2-yl)thiophene-3-carboxylate	CCOC(C1=C(NC(CN(CC(NC2=C(C)C(C)=CC=C2)=O)C)=O)SC(C(C)C)=C1)=O
18	B08	329.347	2-[4-(1-acetamidoethyl)phenyl]-2-oxoethyl 2-methylfuran-3-carboxylate	CC(C1=CC=C(C(COC(C2=C(C)OC=C2)=O)=O)C=C1)NC(C)=O
19	B09	354.356	methyl 2-(3-methyl-4-nitrobenzoyl)-1,2,3,4-tetrahydroisoquinoline-1-carboxylate	COC(C1C2=C(C=CC=C2)CCN1C(C1=CC(C)=C(N(=O)=O)C=C1)=O)=O
20	B10	338.382	2,3-dimethyl-N-[1-(1-methyl-1H-pyrazol-4-yl)ethyl]-6-nitrobenzene-1-sulfonamide	CC(C1=CN(C)N=C1)NS(C1=C(N(=O)=O)C=CC(C)=C1C)(=O)=O
21	C01	349.404	2-(2-methyl-5-nitro-1H-imidazol-1-yl)ethyl 4H,5H,6H,7H,8H-cyclohepta[b]thiophene-2-carboxylate	CC1N(CCOC(C2SC3=C(CCCCC3)C=2)=O)C(N(=O)=O)=CN=1
22	C02	355.344	N-methyl-N-[1-(3-nitrophenyl)ethyl]-3-oxo-3,4-dihydro-2H-1,4-benzoxazine-7-carboxamide	CC(C1=CC(N(=O)=O)=CC=C1)N(C(C1=CC2=C(NC(CO2)=O)C=C1)=O)C
23	C03	346.783	4-chloro-2-fluoro-N-(6-oxo-2-phenylpiperidin-3-yl)benzamide	FC1=C(C(NC2C(C3=CC=CC=C3)NC(=O)CC2)=O)C=CC(Cl)=C1
24	C04	390.817	1-[4-(4-carbamoylphenoxy)benzoyl]pyrrolidine-3-carboxylic acid hydrochloride	NC(C1C=CC(OC2C=CC(C(N3CC(C(=O)O)CC3)=O)=CC=2)=CC=1)=O.Cl
25	C05	366.377	N-(3-methyl-1-phenylbutyl)-2-[2-oxo-5-(trifluoromethyl)-1,2-dihydropyridin-1-yl]acetamide	CC(CC(C1=CC=CC=C1)NC(CN1C(=O)C=CC(C(F)(F)F)=C1)=O)C
26	C06	323.362	N-(butan-2-yl)-2-[2-(3-fluorophenyl)acetamido]butanediamide	CCC(NC(C(NC(CC1=CC(F)=CC=C1)=O)CC(N)=O)=O)C
27	C07	341.405	methyl 5-{[6-(dimethylamino)pyridin-3-yl]sulfamoyl}thiophene-2-carboxylate	COC(C1SC(S(NC2=CN=C(N(C)C)C=C2)(=O)=O)=CC=1)=O
28	C08	387.903	2-({[(5-chloro-2-methylphenyl)carbamoyl]methyl}(ethyl)amino)-N-(3-ethylphenyl)acetamide	CCN(CC(NC1=C(C)C=CC(Cl)=C1)=O)CC(NC1=CC(CC)=CC=C1)=O
29	C09	398.861	2-(5-chlorothiophen-2-yl)-2-oxoethyl 2-{1-methyl-2,4-dioxo-1,3-diazaspiro[4.5]decan-3-yl}acetate	CN1C2(CCCCC2)C(=O)N(CC(OCC(C2SC(Cl)=CC=2)=O)=O)C1=O
30	C10	439.459	4-fluoro-N-[2-oxo-2-(3-oxo-1,2,3,4-tetrahydroquinoxalin-1-yl)ethyl]-N-phenylbenzene-1-sulfonamide	FC1=CC=C(S(N(C2=CC=CC=C2)CC(N2C3=C(C=CC=C3)NC(=O)C2)=O)(=O)=O)C=C1
31	D01	439.385	2-{ethyl[(6-nitro-2,4-dihydro-1,3-benzodioxin-8-yl)methyl]amino}-N-[2-(trifluoromethyl)phenyl]acetamide	CCN(CC1=C2C(COCO2)=CC(N(=O)=O)=C1)CC(NC1=C(C(F)(F)F)C=CC=C1)=O
32	D02	312.430	2-[(2S)-2-(dimethylamino)propanamido]-4-methyl-N-(1,3-thiazol-2-yl)pentanamide	CC(CC(C(NC1SC=CN=1)=O)NC([C@@H](N(C)C)C)=O)C
33	D03	480.597	({3-[(propan-2-yloxy)carbonyl]-4,5,6,7-tetrahydro-1-benzothiophen-2-yl}carbamoyl)methyl 7a-methyl-5-oxo-hexahydropyrrolo[2,1-b][1,3]thiazole-3-carboxylate	CC(OC(C1C2=C(CCCC2)SC=1NC(COC(C1N2C(CCC2=O)(C)SC1)=O)=O)=O)C
34	D04	269.363	2-acetamido-N,3-dimethyl-N-(4-methyl-1,3-thiazol-2-yl)butanamide	CC(C(C(N(C1SC=C(C)N=1)C)=O)NC(C)=O)C
35	D05	343.357	3-(1-phenyl-1H-pyrazole-4-sulfonamido)benzoic acid	OC(C1=CC(NS(C2=CN(C3=CC=CC=C3)N=C2)(=O)=O)=CC=C1)=O
36	D06	390.272	5-bromo-4-[(3,5-dimethylphenyl)sulfamoyl]thiophene-2-carboxylic acid	CC1=CC(NS(C2=C(Br)SC(C(O)=O)=C2)(=O)=O)=CC(C)=C1
37	D07	346.357	1-(3-methyl-4-nitrobenzenesulfonyl)-1,2,3,4-tetrahydroquinolin-4-one	CC1=C(N(=O)=O)C=CC(S(N2C3=C(C=CC=C3)C(=O)CC2)(=O)=O)=C1
38	D08	412.432	N,N-diethyl-4-[2-(4-fluorophenoxy)ethoxy]-3-nitrobenzene-1-sulfonamide	CCN(S(C1=CC(N(=O)=O)=C(OCCOC2=CC=C(F)C=C2)C=C1)(=O)=O)CC
39	D09	395.408	N-[(2H-1,3-benzodioxol-5-yl)methyl]-2-[4-methyl-3-(4-methylphenyl)-2,5-dioxoimidazolidin-1-yl]acetamide	CC1C(=O)N(CC(NCC2=CC3=C(OCO3)C=C2)=O)C(=O)N1C1=CC=C(C)C=C1
40	D10	327.786	4-chloro-N-(2-methanesulfonamido-5-methylphenyl)-1H-pyrrole-2-carboxamide	CC1=CC(NC(C2NC=C(Cl)C=2)=O)=C(NS(C)(=O)=O)C=C1
41	E01	449.498	[(1-methyl-1H-pyrrole-2-carbonyl)carbamoyl]methyl (2S)-3-methyl-2-[2-(naphthalen-1-yl)acetamido]butanoate	CC([C@@H](C(OCC(NC(C1N(C)C=CC=1)=O)=O)=O)NC(CC1=C2C(C=CC=C2)=CC=C1)=O)C
42	E02	393.328	N-(1-cyclopropylethyl)-2-(2,6-dichlorobenzenesulfonamido)-3-methylbutanamide	CC(C(C(NC(C1CC1)C)=O)NS(C1=C(Cl)C=CC=C1Cl)(=O)=O)C

43	E03	469.531	2-[(2-cyclopropyl-3-oxo-2,3-dihydro-1H-isoindol-1-yl)formamido]-N-{[2-(phenoxymethyl)phenyl]methyl}acetamide	O=C(NCC1=C(COC2=CC=CC=C2)C=CC=C1)CNC(C1C2=C(C=CC=C2)C(=O)N1C1CC1)=O
44	E04	371.817	1-[2-(1-benzyl-2,5-dimethyl-1H-pyrrol-3-yl)-2-oxoethyl]-5-chloro-1,2,3,4-tetrahydropyrimidine-2,4-dione	CC1N(CC2=CC=CC=C2)C(C)=C(C(CN2C(=O)NC(=O)C(Cl)=C2)=O)C=1
45	E05	463.548	2-[3-(benzylsulfamoyl)-4-methylbenzoyl]-1,2,3,4-tetrahydroisoquinoline-3-carboxamide	CC1=C(S(NCC2=CC=CC=C2)(=O)=O)C=C(C(N2C(C(N)=O)CC3=C(C=CC=C3)C2)=O)C=C1
46	E06	479.953	2-(5-chlorothiophen-2-yl)-2-oxoethyl 3-[(4-methoxyphenyl)(methyl)sulfamoyl]benzoate	COC1=CC=C(N(S(C2=CC(C(OCC(C3SC(Cl)=CC=3)=O)=O)=CC=C2)(=O)=O)C)C=C1
47	E07	457.279	{[(3-chlorophenyl)methyl]carbamoyl}methyl 2-[(5-chloro-2-fluorophenyl)formamido]-3-hydroxybutanoate	CC(C(C(OCC(NCC1=CC(Cl)=CC=C1)=O)=O)NC(C1=C(F)C=CC(Cl)=C1)=O)O
48	E08	459.560	N-methyl-N-[2-(2-methyl-4-oxo-2,3,4,5-tetrahydro-1H-1,5-benzodiazepin-1-yl)-2-oxoethyl]-4H,5H-naphtho[1,2-b]thiophene-2-carboxamide	CC1N(C(CN(C(C2SC3=C(CCC4=C3C=CC=C4)C=2)=O)C)=O)C2=C(C=CC=C2)NC(=O)C1
49	E09	343.829	N-[(5-chlorothiophen-2-yl)methyl]-2-[2,5-dioxo-4-(propan-2-yl)imidazolidin-1-yl]-N-methylacetamide	CC(C1C(=O)N(CC(N(CC2SC(Cl)=CC=2)C)=O)C(=O)N1)C
50	E10	402.510	N-[(3-{[ethyl(methyl)amino]methyl}phenyl)methyl]-1,3-dimethyl-2-oxo-2,3-dihydro-1H-1,3-benzodiazole-5-sulfonamide	CCN(CC1=CC(CNS(C2=CC3=C(N(C(N3C)=O)C)C=C2)(=O)=O)=CC=C1)C
51	F01	346.404	1-acetyl-N,2-dimethyl-N-(pyrazin-2-yl)-2,3-dihydro-1H-indole-5-sulfonamide	CC1N(C(C)=O)C2=C(C=C(S(N(C3=NC=CN=C3)C)(=O)=O)C=C2)C1
52	F02	357.832	1-{[(4-chlorophenyl)methyl]sulfanyl}-4-methanesulfonyl-2-nitrobenzene	CS(C1=CC(N(=O)=O)=C(SCC2=CC=C(Cl)C=C2)C=C1)(=O)=O
53	F03	435.828	2-[(butan-2-yl)({[2-(1H-1,2,4-triazol-1-yl)-5-(trifluoromethyl)phenyl]carbamoyl}methyl)amino]acetic acid hydrochloride	Cl.CCC(N(CC(NC1C=C(C(F)(F)F)C=CC=1N1N=CN=C1)=O)CC(=O)O)C
54	F04	381.492	5-(2-methyl-1,3-thiazol-4-yl)-N-[2-(2-oxo-1,2-dihydropyridin-1-yl)ethyl]thiophene-2-sulfonamide	CC1SC=C(C2SC(S(NCCN3C(=O)C=CC=C3)(=O)=O)=CC=2)N=1
55	F05	390.840	2-oxooxolan-3-yl 1-[(2-chlorophenyl)methyl]-3-methyl-1H-thieno[2,3-c]pyrazole-5-carboxylate	CC1C2=C(SC(C(OC3C(=O)OCC3)=O)=C2)N(CC2=C(Cl)C=CC=C2)N=1
56	F06	331.333	1-{2-[benzyl(propan-2-yl)amino]acetyl}-3-(2,2,2-trifluoroethyl)urea	CC(N(CC1=CC=CC=C1)CC(NC(NCC(F)(F)F)=O)=O)C
57	F07	374.435	N-[(4R,5S)-1,3-dimethyl-4-(4-methylphenyl)-6-oxo-1H,4H,5H,6H,7H-pyrazolo[3,4-b]pyridin-5-yl]benzamide	CC1C2=C(NC([C@H]([C@@H]2C2=CC=C(C)C=C2)NC(C2=CC=CC=C2)=O)=O)N(C)N=1
58	F08	441.433	N-(6-acetyl-2H-1,3-benzodioxol-5-yl)-2-{3-[2-(cyclohex-1-en-1-yl)ethyl]-2,4,5-trioxoimidazolidin-1-yl}acetamide	CC(C1=C(NC(CN2C(=O)C(=O)N(CCC3CCCCC=3)C2=O)=O)C=C2C(OCO2)=C1)=O
59	F09	318.390	2-(2,5-dimethylbenzenesulfonamido)-2-phenylacetamide	CC1=CC(S(NC(C2=CC=CC=C2)C(N)=O)(=O)=O)=C(C)C=C1
60	F10	405.464	methyl 2-[4-(2,5-dimethylthiophen-3-yl)-4-oxobutanamido]-2-(3-hydroxy-4-methoxyphenyl)acetate	COC(C(C1=CC(O)=C(OC)C=C1)NC(CCC(C1=C(C)SC(C)=C1)=O)=O)=O
61	G01	379.205	3-(3-bromophenyl)-3-[(6-methyl-2-oxo-1,2-dihydropyridin-3-yl)formamido]propanoic acid	CC1NC(=O)C(C(NC(C2C=CC=C(Br)C=2)CC(=O)O)=O)=CC=1
62	G02	341.856	N-[2-(2-chlorophenyl)-2-(dimethylamino)ethyl]-2-(4-oxo-1,3-thiazolidin-3-yl)acetamide	CN(C(C1=C(Cl)C=CC=C1)CNC(CN1C(=O)CSC1)=O)C
63	G03	348.319	(2S)-1-{2-[4-(trifluoromethyl)phenyl]acetyl}-2,3-dihydro-1H-indole-2-carboxamide	NC([C@H]1N(C(CC2=CC=C(C(F)(F)F)C=C2)=O)C2=C(C=CC=C2)C1)=O
64	G04	387.452	N-(3-methyl-1-phenylbutyl)-2,4-dioxo-1,2,3,4-tetrahydroquinazoline-6-sulfonamide	CC(CC(C1=CC=CC=C1)NS(C1=CC2=C(NC(NC2=O)=O)C=C1)(=O)=O)C
65	G05	442.461	[(2H-1,3-benzodioxol-5-yl)carbamoyl]methyl 2-(1,3-dioxo-2,3,3a,4,7,7a-hexahydro-1H-isoindol-2-yl)-4-methylpentanoate	CC(CC(C(OCC(NC1=CC2=C(OCO2)C=C1)=O)=O)N1C(=O)C2C(CC=CC2)C1=O)C
66	G06	278.350	N-(4-cyanophenyl)-5-methylthiophene-2-sulfonamide	CC1SC(S(NC2=CC=C(C#N)C=C2)(=O)=O)=CC=1
67	G07	477.593	2-[1-(1,1-dioxo-1lambda6-thiolan-3-yl)-2,5-dimethyl-1H-pyrrol-3-yl]-2-oxoethyl 4-[4-(methylsulfanyl)phenyl]-4-oxobutanoate	CSC1=CC=C(C(CCC(OCC(C2=C(C)N(C3CS(=O)(=O)CC3)C(C)=C2)=O)=O)=O)C=C1
68	G08	491.562	4-{2-[5-(3,4-dimethoxyphenyl)-3-(thiophen-2-yl)-4,5-dihydro-1H-pyrazol-1-yl]-2-oxoethyl}-2,5-dimethyl-4H,7H-pyrazolo[1,5-a]pyrimidin-7-one	COC1=C(OC)C=C(C2N(C(CN3C4N(N=C(C=4)C)C(=O)C=C3C)=O)N=C(C3SC=CC=3)C2)C=C1
69	G09	341.285	2-(2,4-dioxo-1,2,3,4-tetrahydropyrimidin-1-yl)-N-methyl-N-{[3-(trifluoromethyl)phenyl]methyl}acetamide	CN(C(CN1C(=O)NC(=O)C=C1)=O)CC1=CC(C(F)(F)F)=CC=C1
70	G10	367.420	N,1,3-trimethyl-N-{[5-(2-methylcyclopropyl)furan-2-yl]methyl}-2,4-dioxo-1,2,3,4-tetrahydropyrimidine-5-sulfonamide	CC1C(C2OC(CN(S(C3C(=O)N(C)C(=O)N(C)C=3)(=O)=O)C)=CC=2)C1
71	H01	425.861	N-(2-chlorophenyl)-2-(N-methyl7-fluoro-2-oxo-1,2,3,4-tetrahydroquinoline-6-sulfonamido)acetamide	CN(S(C1=C(F)C=C2C(CCC(N2)=O)=C1)(=O)=O)CC(NC1=C(Cl)C=CC=C1)=O
72	H02	353.457	2-[methyl({[(4-methylphenyl)carbamoyl]methyl})amino]-N-[1-(2-methylphenyl)ethyl]acetamide	CC(C1=C(C)C=CC=C1)NC(CN(CC(NC1=CC=C(C)C=C1)=O)C)=O
73	H03	350.434	N-(4-methylphenyl)-2-{5-[(3-methylphenyl)methylidene]-4-oxo-1,3-thiazolidin-2-ylidene}acetamide	CC1=CC=C(NC(C=C2SC(=CC3=CC(C)=CC=C3)C(=O)N2)=O)C=C1
74	H04	324.352	methyl 4-{N-[(3-methyl-1,2-oxazol-5-yl)methyl]methanesulfonamido}benzoate	COC(C1=CC=C(N(S(C)(=O)=O)CC2ON=C(C)C=2)C=C1)=O
75	H05	329.368	2-{2-[(4-fluoro-3-methylphenyl)amino]acetamido}-N-methyl-2-phenylacetamide	CNC(C(C1=CC=CC=C1)NC(CNC1=CC(C)=C(F)C=C1)=O)=O
76	H06	332.781	4-[3-(benzyloxy)propanamido]-2-chlorobenzamide	NC(C1=C(Cl)C=C(NC(CCOCC2=CC=CC=C2)=O)C=C1)=O
77	H07	412.458	2-[5-(2-acetamidoethyl)thiophen-2-yl]-2-oxoethyl 5-methyl-3-phenyl-1,2-oxazole-4-carboxylate	CC(NCCC1SC(C(COC(C2C(C3=CC=CC=C3)=NOC=2C)=O)=O)=CC=1)=O
78	H08	330.358	methyl 3-[(2-cyanophenyl)methanesulfonamido]benzoate	COC(C1=CC(NS(CC2=C(C#N)C=CC=C2)(=O)=O)=CC=C1)=O
79	H09	466.572	6-[6,7-dimethoxy-1-(thiophen-2-yl)-1,2,3,4-tetrahydroisoquinoline-2-carbonyl]-3,4-dihydro-2H-1,4-benzothiazin-3-one	COC1=C(OC)C=C2C(CCN(C(C3=CC4=C(SCC(N4)=O)C=C3)=O)C2C2SC=CC=2)=C1
80	H10	417.766	N-[4-chloro-2-(trifluoromethyl)phenyl]-2-(3-cyclopentyl-2,4,5-trioxoimidazolidin-1-yl)acetamide	FC(C1=C(NC(CN2C(=O)C(=O)N(C3CCCC3)C2=O)=O)C=CC(Cl)=C1)(F)F
81	I01	329.345	N-[2-(difluoromethyl)-1H-1,3-benzodiazol-5-yl]thiophene-2-sulfonamide	FC(C1NC2=C(C=C(C=C2)NS(C2SC=CC=2)(=O)=O)N=1)F
82	I02	308.377	2-[2-methyl-4-(4-methylphenyl)-3,4-dihydroquinazolin-3-yl]acetohydrazide	CC1N(CC(NN)=O)C(C2=CC=C(C)C=C2)C2=C(C=CC=C2)N=1
83	I03	447.289	4-{4-[(2,4-dichlorophenyl)methoxy]-3-nitrobenzenesulfonyl}morpholine	O=N(C1=C(OCC2=C(Cl)C=C(Cl)C=C2)C=CC(S(N2CCOCC2)(=O)=O)=C1)=O

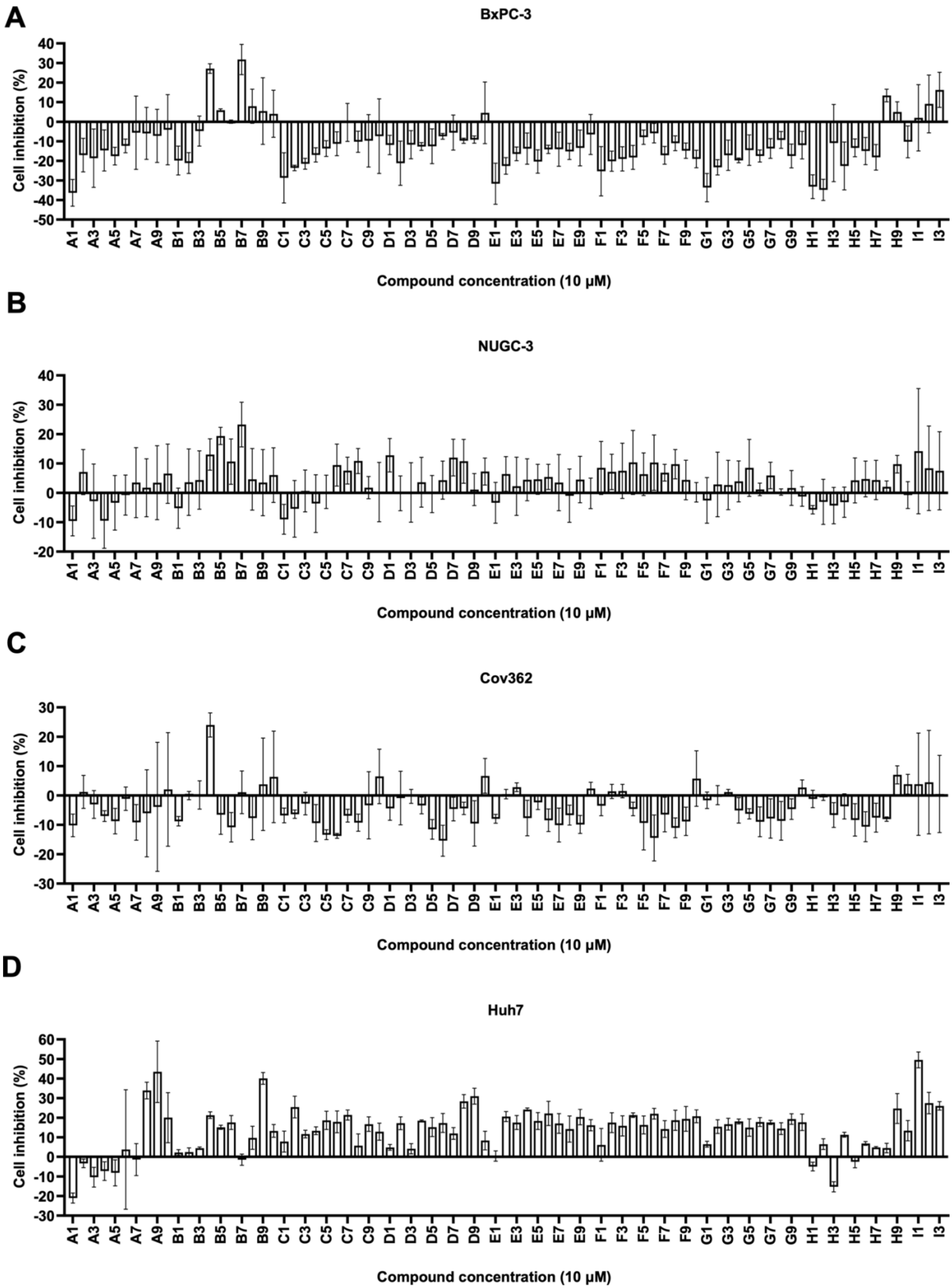
A
BxPC-3
Cell inhibition (%)
Compound concentration (10 µM)
B
NUGC-3
Cell inhibition (%)
Compound concentration (10 µM)
C
Cov362
Cell inhibition (%)
Compound concentration (10 µM)
D
Huh7
Cell inhibition (%)
Compound concentration (10 µM)

Figure S1 Compound screening. Cell viability, normalized to DMSO controls, of BxPC-3 (A), NUGC-3 (B), Cov362 (C), and Huh7 (D) cells treated for 72 h with indicated compounds. Error bars in (A-D) represent the means ± SD.

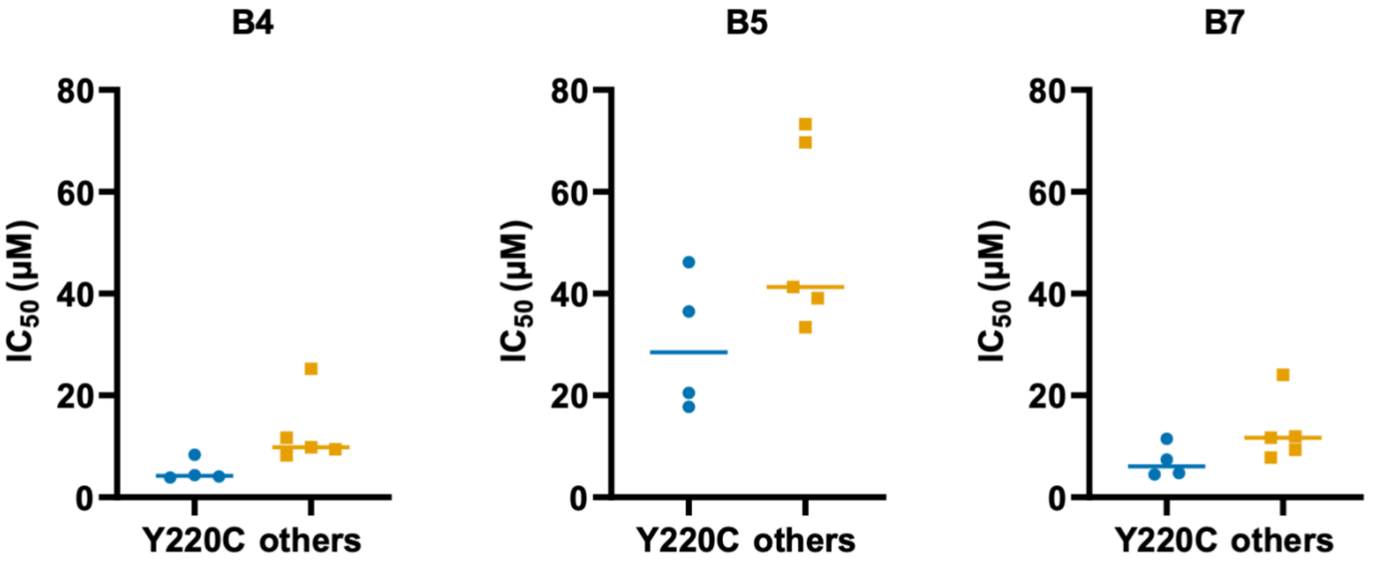

Figure S2 Y220C specificity of compounds in inhibiting cell proliferation. The dots represent the calculated IC_{50} in cell lines with *p53-Y220C* mutation or other mutations.

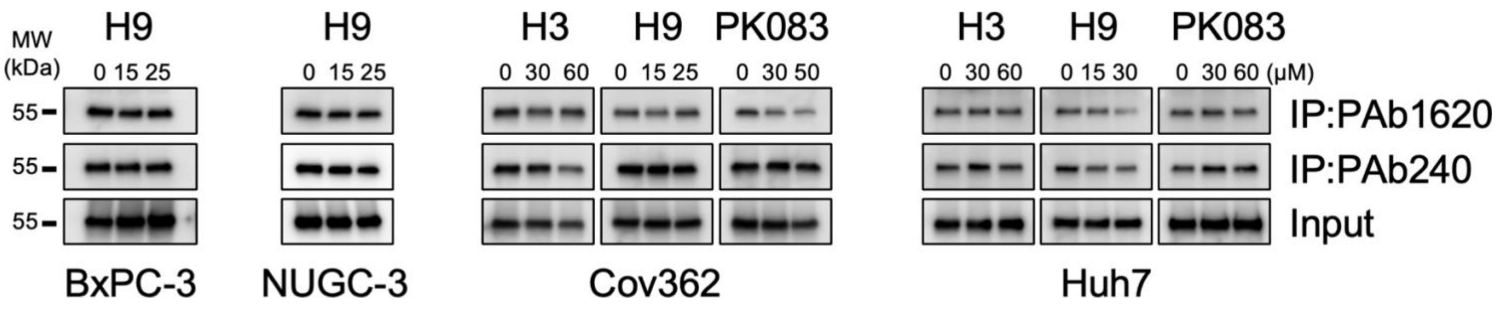

Figure S3 Structural conversion induced by H3 and H9. PA1620 and PAb240 IP for cells expressing endogenous p53-Y220C mutant treated with H3, H9, and PK083 for 24 h. Immunoblotting was performed using the anti-p53 DO-1 antibody after IP.

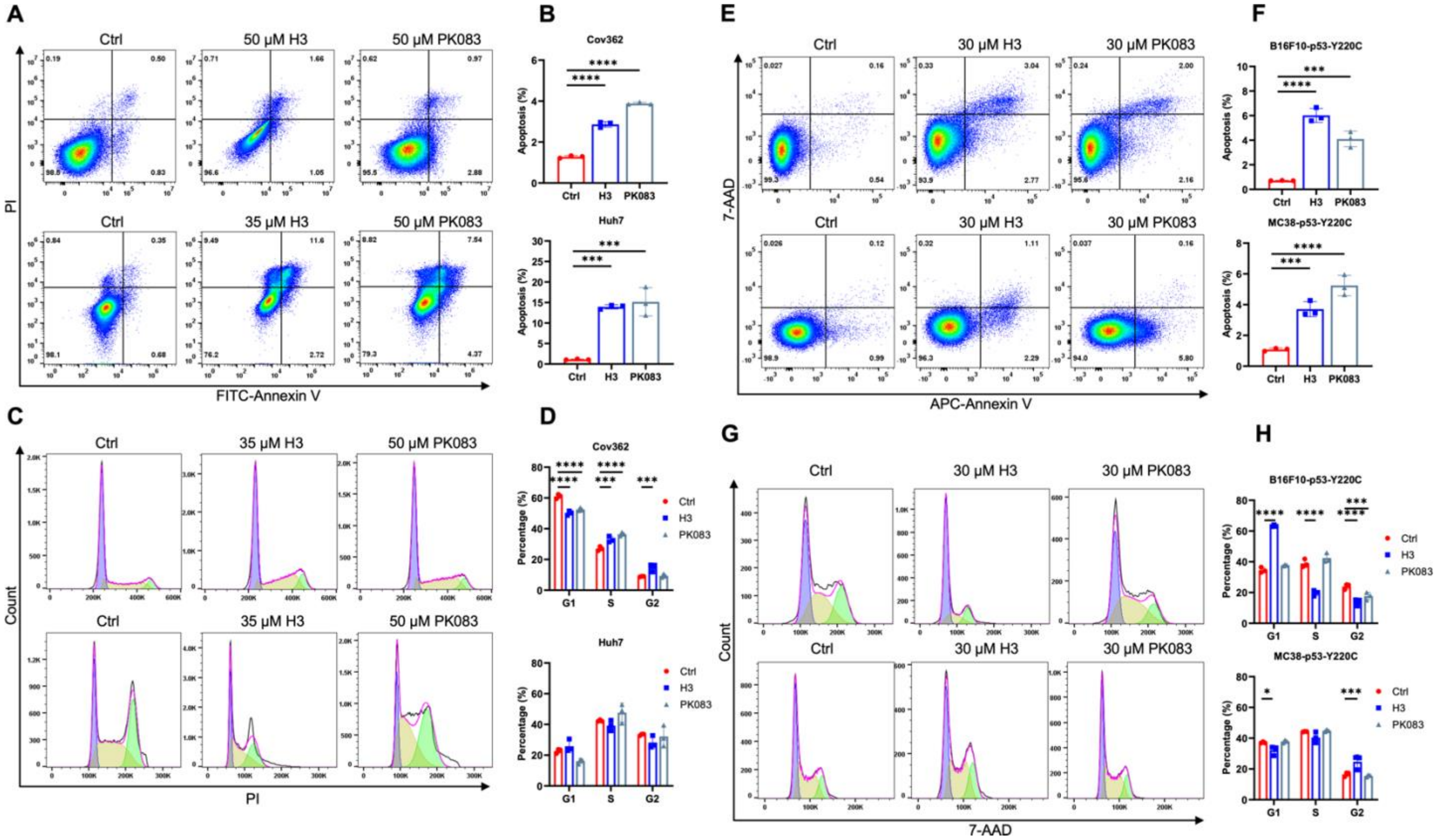

Figure S4 H3 induces cycle arrest and apoptosis in p53-Y220C mutant cells. (A) Flow cytometric plots of Annexin V vs. PI staining in Cov362 cells after treatment with 50 μM H3 or PK083 for 48 h, and Huh7 cells after treatment with 35 μM H3 or 50 μM PK083 for 24 h. (B) From the histograms, the proportion of cell apoptosis were statistically analyzed. *p* values were calculated by one-way ANOVA followed by Dunnett's multiple-comparison test. (C) Cell cycle analysis by flow cytometry after PI staining of Cov362 cells treated with 35 μM H3 or 50 μM PK083 for 24 h or Huh7 cells treated with 35 μM H3 or 50 μM PK083 for 24 h. (D) From the histograms, the proportion of cell apoptosis and cell cycle distribution were statistically analyzed. *p* values were calculated by two-way ANOVA followed by Dunnett's multiple-comparison test. (E) Flow cytometric plots of Annexin V vs. 7-AAD staining in B16F10-p53-Y220C and MC38-p53-Y220C cells after treatment with 30 μM H3 or 30 μM PK083 for 24 h. (F) From the histograms, the proportion of cell apoptosis were statistically analyzed. *p* values were calculated by one-way ANOVA followed by Dunnett's multiple-comparison test. (G) Cell cycle analysis through 7-AAD staining and following flow cytometry. B16F10-p53-Y220C and MC38-p53-Y220C cells after treatment with 30 μM H3 or 30 μM PK083 for 24 h. (H) From the histograms, the proportion of cell cycle distribution were statistically analyzed. *p* values were calculated by

two-way ANOVA followed by Dunnett's multiple-comparison test. *$p < 0.05$, **$p < 0.01$, ***$p < 0.001$, and ****$p < 0.0001$. Error Bars in (B, D, F and H) represent mean ± SD.

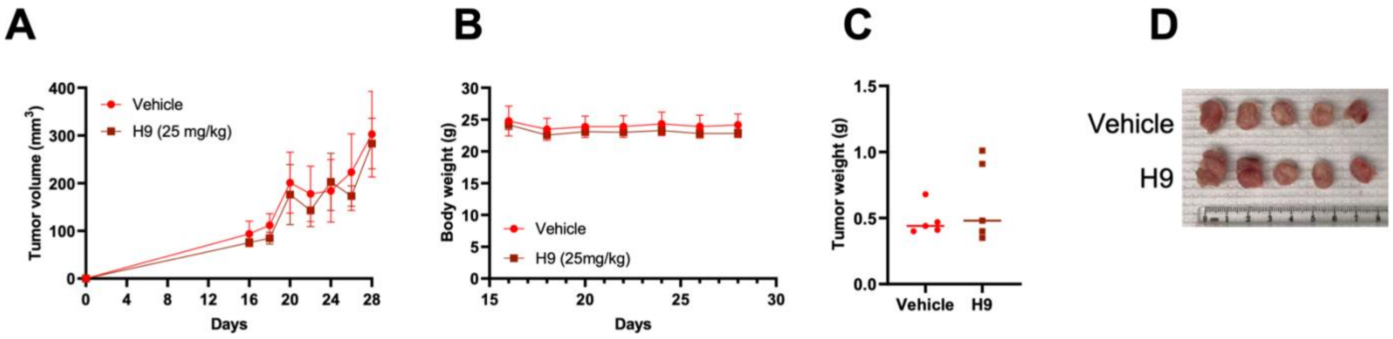

Figure S5 H9 administration on NSG mice. (A) Immunodeficient NSG mice were injected with NUGC-3 cells on day 0. Sixteen days post-inoculation, ten mice were randomly divided into two groups, then mice were treated with 25 mg/kg H9 every other day until the first control mouse reached humane endpoints. Tumor size was measured every other day. (B) Body weight of mice carrying the NUGC-3 tumors after treatments. (C) Tumors were weighed at the end of the experiment. (D) Images of the tumors excised from the mice on day 28 in the various treatment groups. Error Bars in (A) and (B) represent mean ± SD. *p* values were calculated by a two-tailed *t*-test.
